# Supplementary material for: Multi-Scale Digital Pathology Patch-Level Prostate Cancer Grading Using Deep Learning: Use Case Evaluation of DiagSet Dataset
Source: Bioengineering (Basel). 2024 Jun 18;11(6):624. doi: 10.3390/bioengineering11060624 (PMC11200755; doi:10.3390/bioengineering11060624)
Supplement: Supplementary file 1 [file bioengineering-11-00624-s001.zip › bioengineering-3021527-supplementary.pdf]

bioengineering

MDPI

Supplementary Materials

Multi-Scale Digital Pathology Patch-Level Prostate Cancer Grading Using Deep Learning: Use Case Evaluation of DiagSet Dataset

Tanaya Kondejkar [1], Salah Mohammed Awad Al-Heejawi [1], Anne Breggia [2], Bilal Ahmad [3], Robert Christman [3], Stephen T. Ryan [3] and Saeed Amal [4,*]

[1] College of Engineering, Northeastern University, Boston, MA 02115, USA; kondejkar.t@northeastern.edu (T.K.); s.al-heejawi@northeastern.edu (S.M.A.A.-H.)
[2] MaineHealth Institute for Research, Scarborough, ME 04074, USA; anne.breggia@mainehealth.org
[3] Maine Medical Center, Portland, ME 04102, USA; bilal.ahmad@spectrumhcp.com (B.A.); robert.christman@spectrumhcp.com (R.C.); stephen.ryan@mainehealth.org (S.T.R.)
[4] The Roux Institute, Department of Bioengineering, College of Engineering, Northeastern University, Boston, MA 02115, USA
* Correspondence: s.amal@northeastern.edu

Resnet34 on all magnifications:

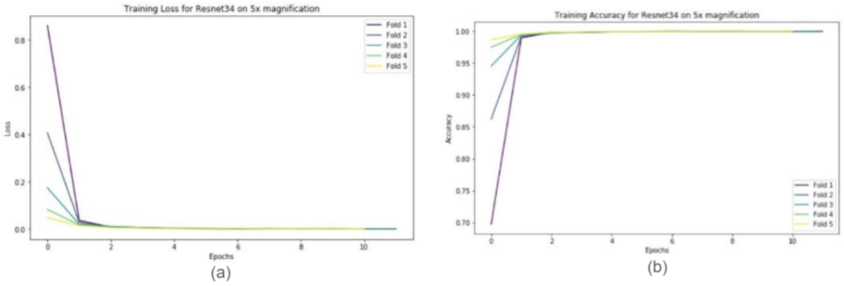

Figure S1. Training loss (**a**) and training accuracy (**b**) for Resnet34 on 5× magnification.

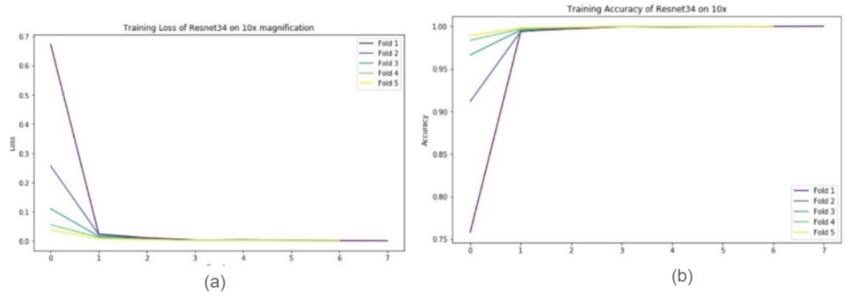

Figure S2. Training loss (**a**) and training accuracy (**b**) for Resnet34 on 10× magnification.

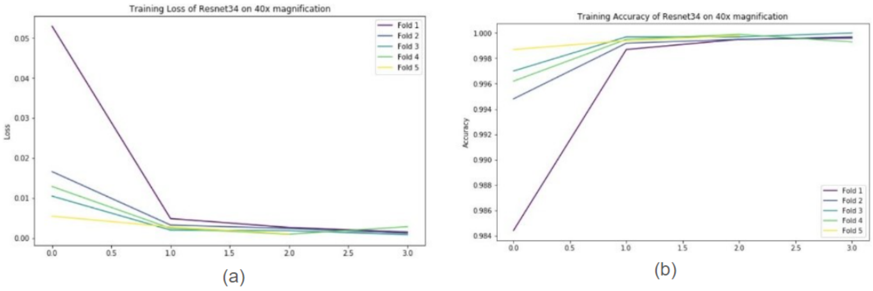

Figure S3. Training loss (**a**) and training accuracy (**b**) for Resnet34 on 40× magnification.

Resnet18 on all magnifications:

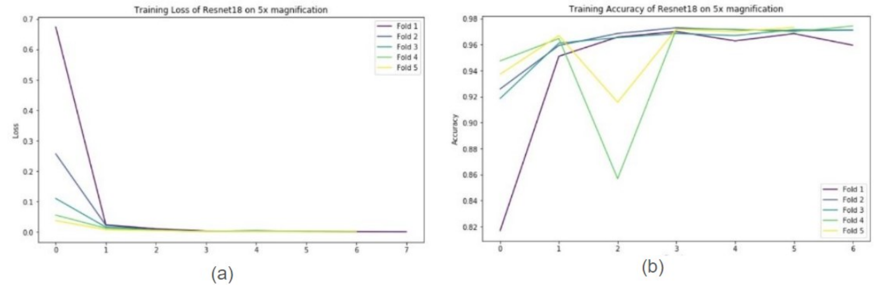

Figure S4. Training loss (**a**) and training accuracy (**b**) for Resnet18 on 5× magnification.

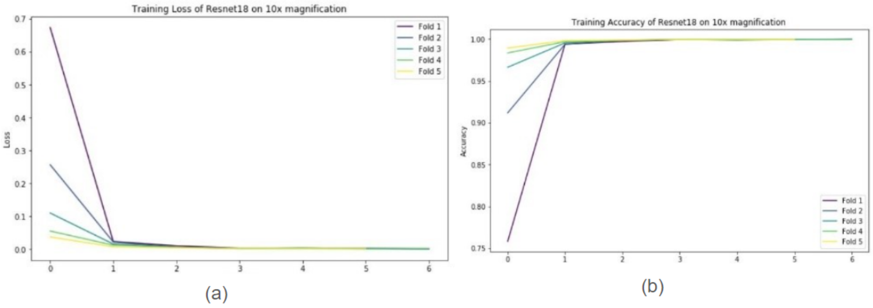

Figure S5. Training loss (**a**) and training accuracy (**b**) for Resnet18 on 10× magnification.

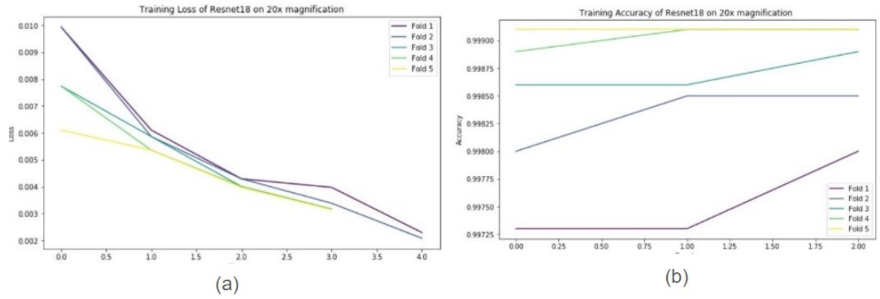

Figure 6. S Training loss (**a**) and training accuracy (**b**) for Resnet18 on 20× magnification.

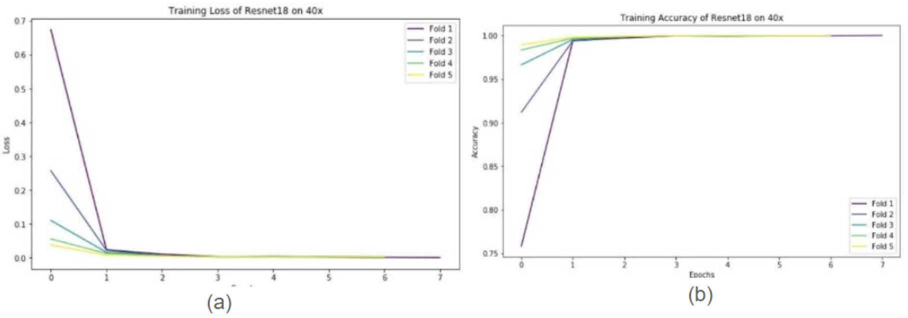

Figure 7. STraining loss (**a**) and training accuracy (**b**) for Resnet18 on 40× magnification.

Resnet50 Training Loss and Accuracy across all magnifications:

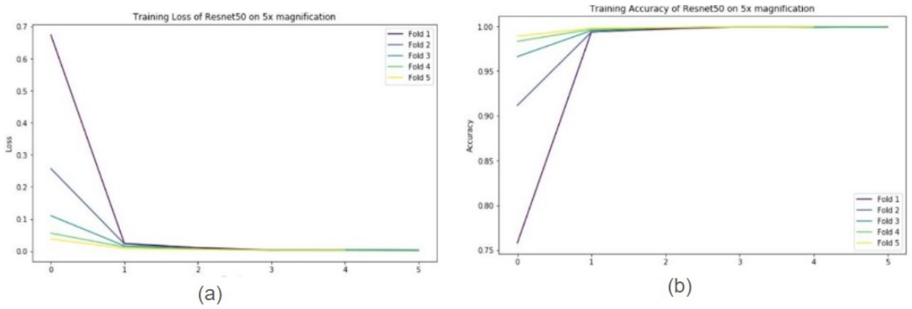

Figure S8. Training loss (**a**) and training accuracy (**b**) for Resnet50 on 5× magnification.

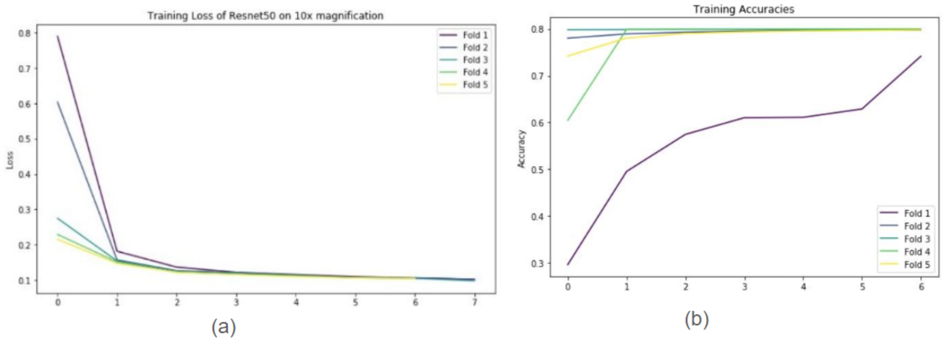

Figure S9. Training loss (**a**) and training accuracy (**b**) for Resnet50 on 10× magnification.

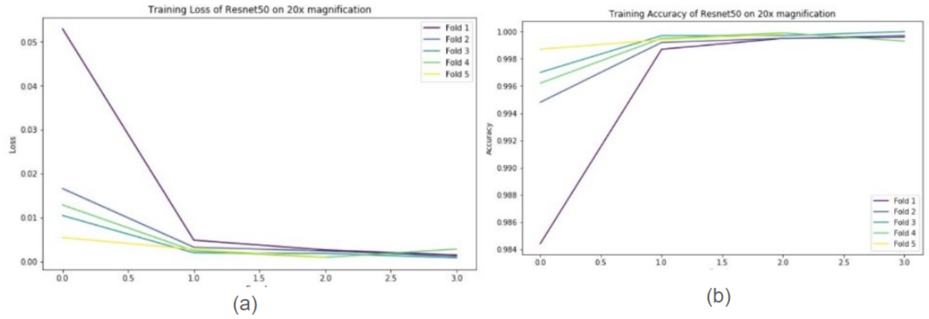

Figure S10. Training loss (**a**) and training accuracy (**b**) for Resnet50 on 20× magnification.

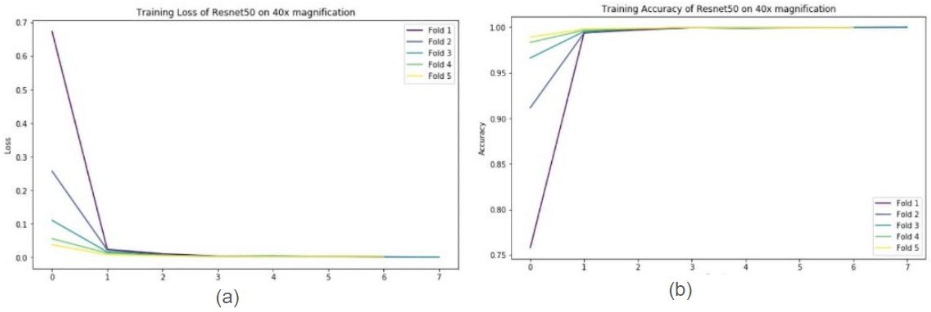

Figure S11. Training loss (**a**) and training accuracy (**b**) for Resnet50 on 40x magnification.
